# Trajectories of reproductive transition phase mood disorder from pregnancy to postpartum: A Swiss longitudinal study

**DOI:** 10.1177/17455057221147391

**Published:** 2023-02-07

**Authors:** Alexandra Johann, Jelena Dukic, Yannick Rothacher, Ulrike Ehlert

**Affiliations:** 1 Clinical Psychology and Psychotherapy, University of Zurich, Zurich, Switzerland; 2 Psychological Methods, Evaluation and Statistics, University of Zurich, Zurich, Switzerland

**Keywords:** mood disorder, postpartum depression, pregnancy, symptomatology, trajectories

## Abstract

**Background:**

Depressive symptoms are common in the peripartum period and pose a great risk to the well-being of the mother, the infant, and the entire family. Evidence from longitudinal studies suggests that affected women do not constitute one homogeneous group in terms of severity, chronicity, and onset of symptoms. To account for individual differences regarding the longitudinal course of depressive symptoms from pregnancy to the postpartum period, growth mixture models have proven to be useful.

**Methods:**

We conducted a group-based trajectory modeling analysis to identify perinatal depressive symptom trajectories in a Swiss sample (*n* = 151). Depressive symptoms were assessed six times, covering nearly 6 months from the third trimester of pregnancy to 3 months postpartum. In addition to determining perinatal depressive symptom trajectories, we aimed to examine whether these trajectories are linked to psychopathological risk factors such as a history of premenstrual syndrome (PMS), anxiety, prenatal stress, and somatic symptoms after delivery that are associated with hormonal fluctuations.

**Results:**

The findings revealed three trajectories of perinatal depressive symptoms that were relatively stable over time and differed in symptom load (low, medium, high), as well as one trajectory of decreasing symptoms, with a significant symptom reduction after giving birth. Women with a higher depressive symptom load experienced a greater degree of prior premenstrual symptoms, prenatal anxiety, and birth anxiety, as well as somatic symptoms after delivery.

**Conclusion:**

Further research is needed to account for the distinct trajectories of perinatal depressive symptoms in order to provide appropriate care for affected women. A focus on somatic symptoms after delivery and their association with depressive mood is essential to better understand the potential shared etiopathology of reproductive transition phase mood disorders.

## Introduction

Mood disorders during pregnancy and following childbirth represent one of the major complications in the perinatal period and result in a vast burden on global health. Perinatal depressive symptoms pose a threat not only to the affected individual’s mental health and quality of life but also to the physical and mental health of the offspring, partner, and entire family.^1,2^ In a large, mainly Swiss sample (*n* = 687) from a longitudinal study conducted by our research group, we found prevalence rates of 18.3% for antenatal depression and 18.5% for postpartum depressive symptoms. Evidence shows that an extensive number of postpartum depressive symptoms first emerge during pregnancy and continue after childbirth and beyond.^3^ For instance, in a longitudinal study, a quarter of mothers from a population-based birth cohort continued to have elevated depressive symptoms 3 years postpartum.^4^ To date, only a limited number of studies have captured the evolution of depressive symptoms in the perinatal period longitudinally. These studies demonstrated that depressive symptom profiles during pregnancy and the postpartum period seem to be heterogeneous regarding the characteristics and chronicity of symptoms.^4–15^

Our systematic review of the literature on the clinical presentation of perinatal depressive symptoms revealed a higher prevalence of atypical symptoms, possibly linked to the unique physiological and psychological changes during the puerperium.^16^ The most common comorbidities with depressive symptoms were anxiety and somatic symptoms, which contributed further to the psychopathological burden.^16^ In addition to depressive symptoms, anxiety has been found to be highly prevalent in perinatal women, especially among those with a history of depression and pregnancy-related stress.^13,15^ A longitudinal study of perinatal women in China (*n* = 412) reported that the onset and chronicity of depressive symptoms postpartum was associated with poor sleep trajectories prenatally.^12^

The longitudinal studies conducted to date have mainly focused on demographic, psychosocial, and birth-related factors or child outcome measures.^6,8–10,17^ For example, chronically high depressive symptoms during the peripartum have been associated with more pregnancy-related distress and social conflict, as well as lower levels of social support.^14^ However, perinatal depressive symptoms seem to be affected by a variety of psychopathological risk factors such as anxiety, somatic symptoms, history of depression, or premenstrual syndrome (PMS).^5,10,18–20^

There is strong evidence that reproductive transition phases—such as the luteal phase of the menstrual cycle, the peripartum period, and the perimenopause—are especially vulnerable time windows for the development of mood disorders in some women. The literature suggests that this heightened vulnerability is likely due to failed adaptation to the inherent hormonal, inflammatory, and psychosocial alternations during reproductive transition phases.^11,21^ For instance, women who show a greater vulnerability to develop depressive symptoms during pregnancy and the postpartum phase are more likely to have a history of PMS and a greater risk for mood disorders during reproductive transition phases.^18,20,22,23^

The onset of somatic symptoms such as hot flashes and urogenital complaints, which typically occur during and after the menopausal transition, has also been described in the perinatal period.^24,25^ The delivery of the placenta after childbirth triggers a rapid decline in estrogen and progesterone in the early postpartum period. However, this hormonal withdrawal after delivery is much sharper and of shorter duration compared to the perimenopause. It has been hypothesized that depressive symptoms throughout reproductive transition phases are associated with somatic symptoms due to hormonal withdrawal and/or fluctuations.^16,26,27^ Given that these symptoms—such as hot flashes, urogenital complaints, and disrupted sleep—occur during all reproductive transition phases, a shared etiopathology has been assumed,^28^ although perinatal sleep disruptions most likely have multifactorial causes, such as pregnancy-related pain or caring for the newborn after delivery. A recent study found a significant treatment response to transdermal estradiol in perimenopausal women with a high estradiol sensitivity, resulting in fewer anxiety and somatic symptoms.^29^ Interestingly, the occurrence of such somatic symptoms associated with hormonal withdrawal and/or fluctuations, as a potential factor associated with postpartum depressive symptoms, has not yet been evaluated. Moreover, to date, there is no questionnaire to assess somatic symptoms during all reproductive transition phases, despite the significant overlap on the symptom level.^24,28,30,31^ In general, clinical indications regarding specific characteristics of depressive symptoms during reproductive transition phases, and therefore potential psychopathological predictors, remain to be identified.

It is important to point out that the majority of psychopathological predictors of perinatal depressive symptoms—such as somatic symptoms, anxiety, and a history of PMS—were validated in cross-sectional studies.^17,23,32,33^ Therefore, the potential predictive value of psychopathological risk factors linked to different trajectories of perinatal depressive symptoms is still limited due to a lack of longitudinal research.

This study seeks to examine the presence of heterogeneous depressive symptom trajectories from pregnancy to postpartum in a Swiss sample, and to determine whether psychopathological risk factors are associated with unique longitudinal symptom patterns. The aim is to evaluate whether certain psychopathological factors increase the risk of perinatal depressive symptoms in specific subgroups of women. Such factors might, for instance, be a history of premenstrual symptoms or the incidence of somatic symptoms associated with hormonal fluctuations, which might render a specific subgroup of women more vulnerable to develop depressive symptoms in the reproductive transition phase from pregnancy to postpartum. The ultimate goal is to improve the screening and diagnosis of the most vulnerable women in order to prospectively improve treatment by providing timely interventions.

## Methods

This study was part of a large research project funded by the Swiss National Science Foundation, carried out at the University of Zurich, Department of Clinical Psychology and Psychotherapy. The research project consisted of an observational, single-center, longitudinal study with a mean duration of 17 weeks per participant. Physically healthy, pregnant women were included in the study. Participants either had a history of depression, current depressive symptoms, or no current or past depressive symptoms. We chose this sample in order to identify whether symptom profiles might differ according to women’s prior or current depression status. Data were collected between June 2019 and June 2021. Participants were first contacted during their third trimester of pregnancy, at approximately 34–36 weeks gestation, and were followed up until 8–12 weeks postpartum. A variety of (epi-)genetic, biological, physiological, and psychological parameters were assessed within the research project; detailed information can be found in the study protocol.^34^ The large research project was approved by the Ethics Committee of the Canton of Zurich (KEK-ZH-Nr. 2018–02357) and conducted in accordance with the Declaration of Helsinki.

### Participants

Physically healthy, pregnant women were recruited in Zurich, Switzerland, and the surrounding area through pregnancy-related websites, social media (Facebook, Instagram), birth clinics, obstetricians, and antenatal classes. The aim was to investigate women with a history of depression, women with current depressive symptoms, and women with no history of depression/no current depressive symptoms. In order to reach currently depressed women and encourage them to participate, we offered an incentive of 10 free-of-charge psychotherapy sessions at our outpatient clinic. If women expressed interest in the psychotherapy sessions, they were contacted after completion of study participation in order to prevent any effect on the study results. To exclude any confounding comorbidities, women were screened with the German version of the Structured Clinical Interview for the *Diagnostic and Statistical Manual of Mental Disorders* (4th ed.; DSM-IV)^35^ upon enrollment.

The following exclusion criteria were applied: multifetal gestation or pregnancies achieved through assisted reproductive technology; any medical complications (e.g. hypertension, diabetes mellitus, hyperemesis gravidarum, (pre)eclampsia, suspected fetal growth restriction, fetal structural abnormalities) or medical conditions that might have affected ovarian function prior to pregnancy (e.g. polycystic ovary syndrome, endometriosis, breast cancers); and current or past psychosis, bipolar disorder, posttraumatic stress disorder, eating disorder, and substance abuse or dependency. Moreover, to eliminate any hormonal confounding variables, women with current intake of hormones (e.g. corticosteroids), diuretics, hypertensives, or vasodilators were excluded. Finally, treatment with psychotropic substances within the last 3 months prior to study inclusion, drug use and/or smoking, alcohol intake of more than one standard unit a day, pre-pregnancy body mass index (BMI) >25 or <18, a protein-restricted diet, and/or regular consumption of soy products led to exclusion from the study.

For the sample size estimation, we performed an a priori sample size estimation analysis using GPower 3.1.^36^ The analysis was computed considering a medium effect size of 0.25 with *α* = 0.05 and power = 0.95 (1 − *ßa*). As only pregnant women who went on to give birth to a healthy child were to be included in the statistical analyses, and assuming a dropout rate of approximately 10%, a sample size of *N* = 288 was originally estimated. However, the recruitment process was greatly affected by the ongoing COVID-19 pandemic, and the sample size had to be adjusted accordingly after recruiting for 2 years. The original sample size estimation was based on the overall large research project described above, including all variables examined in the project. Due to the lack of pre-existing literature regarding post hoc analysis of group-based trajectory modeling, we focused on the recommended quality criteria to achieve the most parsimonious model fit for the present data.

**Figure 1.**
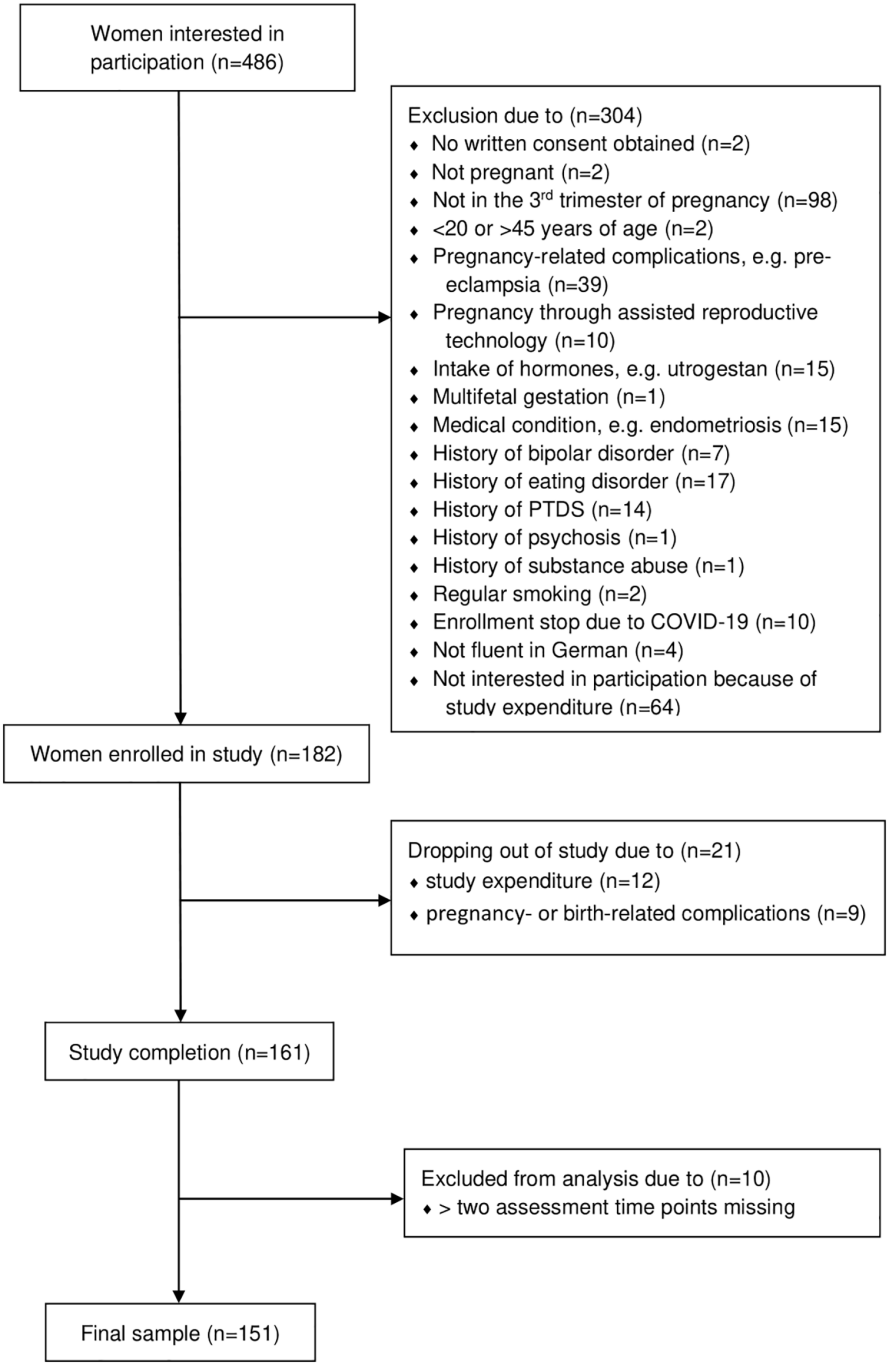
Sample inclusion. EPDS: Edinburgh Postnatal Depression Scale; STAI: State-Trait Anxiety Inventory; BAS: Birth Anxiety Scale; PMS: premenstrual syndrome; PDQ: Prenatal Distress Questionnaire; MRS: Menopause Rating Scale; pp: postpartum.

A total of 182 women were included, of whom 161 ultimately completed the study procedure. Women who dropped out of the study after enrollment were mostly Swiss, in a long-term relationship, still working, primiparous, with a university degree, not in current psychiatric–psychotherapeutic care, and without a history of depression. Reasons for dropout were mainly preterm birth, complications during delivery, and the time expenditure associated with study participation. Within the following analyses, the sample sizes vary on account of scattered missing data; only participants with almost complete data sets (maximum two assessment time points missing) were included in the final analyses (*n* = 151). The study inclusion procedure is presented in Figure 1.

### Procedure

**Figure 2.**
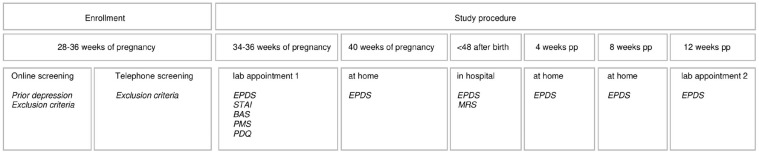
Study procedure.

Before enrollment, interested women were screened for eligibility using an online questionnaire (*n* = 486). If potential participants were deemed to be eligible and provided written consent, a telephone interview was conducted to confirm the inclusion criteria and finalize enrollment. At 34–36 weeks gestation, women were invited to a lab appointment at the University of Zurich, Department of Clinical Psychology and Psychotherapy. During this first lab session, various psychological, biological, and (epi-) genetic parameters were assessed (an overview and more detailed information are provided in the Study Protocol).^34^ At the end of the lab appointment, the women were instructed about the follow-up measurements to be taken at home. The second and final lab appointment was scheduled around 12 weeks after giving birth and assessed the same parameters as during pregnancy as well as birth-related information. An overview of the study procedure and assessment time points can be found in Figure 2.

### Measures

All relevant psychological variables in this study were assessed using validated German versions of self-report questionnaires. Before completing the questionnaires, participants were required to provide written consent regarding study enrollment in order to proceed. Online data were gathered using the Unipark platform (www.unipark.com/de, certificate ISO 27001). This reliable, BSI-certified data center fulfills the requirements of high data protection and safety in conformity with ISO 27001.

#### History of depression

First, history of depression was assessed during the online screening, referring to a previous diagnosis of depression or self-report of prior depression, operationalized as yes/no. Second, to avoid missing cases, participants were asked about past depressive symptoms by presenting them with a list of the criteria for major depression according to the *Diagnostic and Statistical Manual of Mental Disorders* (5th ed.; DSM-5). If women confirmed three or more depressive symptoms in the past, they were included in the prior depression group.

#### Depressive symptoms

At the first lab visit and at all subsequent assessments, participants completed the German version of the Edinburgh Postnatal Depression Scale (EPDS) to assess depressive symptoms during pregnancy and the postpartum period. The EPDS is a validated, widely used screening tool for postpartum depression.^37^ It consists of 10 self-report items and has shown satisfactory specificity and sensitivity. The original cut-off score to identify a clinically relevant episode of major depression is 12/13.^37^ Recently, it has been suggested that a categorical approach would allow for a more detailed distinction of symptom severity: minimal depression (0–6), mild depression (7–13), moderate depression (14–19), and severe depression (19–30).^38^ The reliability analysis in the original validation of the German version reported *α* = 0.81.^39^ The mean Cronbach’s alpha in this study, measured across six time points, was *α* = 0.86.

#### Birth anxiety

Anxiety regarding various birth-related aspects, such as the delivery itself or birth-related complications, was assessed using the Birth Anxiety Scale (BAS).^40^ The BAS is a self-report scale consisting of 25 items rated on a 4-point Likert-type scale ranging from “that does not scare me at all” to “that scares me a lot.” Internal consistency of the scale in this study was high (*α* = 0.89).

#### State anxiety

State anxiety was operationalized using the subscale for state anxiety from the German version of the State-Trait Anxiety Inventory.^41^ The scale comprises 20 self-report items that are rated on a 4-point Likert-type scale. Internal consistency for the state subscale in the original validation study lay at *α* = 0.90; internal consistency in this study was *α* = 0.91.

#### Premenstrual symptoms

A history of premenstrual symptoms was assessed using the German PMS inventory.^42^ This self-report inventory was developed based on the *Diagnostic and Statistical Manual of Mental Disorders* (4th ed., text rev.; DSM-IV-TR) criteria for Premenstrual Dysphoric Disorder (PMDD) and captures somatic and psychological symptoms during the luteal phase of the menstrual cycle. The occurrence and severity of premenstrual symptoms are assessed through 30 items rated on a 4-point scale. Internal consistency in the original validation study lay at *α* = 0.88; internal consistency in the present sample was *α* = 0.93.

#### Somatic symptoms after delivery

The transition from pregnancy to the postpartum period is strongly associated with hormonal alterations. In particular, the hormonal withdrawal after delivery can manifest in symptoms ranging from psychological to somatic symptoms. The sharp drop in estrogen and progesterone after delivery is similar to the sex steroid withdrawal during the perimenopause or the luteal phase of the menstrual cycle, although of much greater extent and shorter duration. During the perimenopause, these symptoms have been strongly linked to the development of depressive symptoms.^26^ To assess the occurrence of these somatic symptoms in the postpartum period, we applied the German version of the Menopause Rating Scale-II (MRS-II),^43^ due to the lack of an equivalent questionnaire for somatic symptoms postpartum. Women rated their symptoms immediately after giving birth (<48 h), as well as 4, 8, and 12 weeks after delivery. The Menopause Rating Scale is a valid, self-report 11-item scale to rate the presence and severity of menopausal symptoms. For ease of reading, in the following, we will refer to these somatic symptoms after delivery as quasi-menopausal symptoms. The items can be clustered into three subscales that assess psychological, somatic-vegetative, and urogenital symptoms. In the original study, internal consistency was reported to be *α* = 0.83;^44^ overall Cronbach’s alpha in this study lay at *α* = 0.71.

#### Prenatal stress

To assess prenatal stress, we administered the German version of the Prenatal Distress Questionnaire (PDQ).^45^ Specifically, women were asked how concerned they are about certain pregnancy-related topics, with items rated on a 4-point Likert-type scale from “not at all” to “extremely.” Cronbach’s alpha was *α* = 0.81 in the original validation study and *α* = 0.79 in this study.

### Statistical analysis

To evaluate the longitudinal course of symptoms, different statistical approaches are established. In contrast to standard growth curve modeling, which is usually based on a single average trajectory of symptoms, group-based trajectory modeling (GBTM) enables multiple trajectories to be located within a sample.^46^ Specifically, GBTM is a subtype of growth mixture modeling that detects subgroups of individuals who share a similar pattern of symptoms over time, and was thus used in this study to analyze the occurrence of distinct trajectories of perinatal depressive symptoms in mentally healthy women, women with a history of depression, and women with current depression.^47^ In addition to identifying different trajectories of symptom patterns, we sought to determine potential distinguishing factors that define group membership. To model the trajectories of perinatal depressive symptoms and to identify predictors that define group affiliation, the lcmm package in R was employed.^48^ This function fits latent class linear mixed models (LCLMMs), presuming that the population under study can be partitioned into a finite number of latent classes.^49^ Class membership of each individual, described here as group affiliation, and the trajectory itself are defined by covariates or predictors.^49^ Regarding best model fit in terms of the number and shape of trajectory groups, the recommended selection criteria such as the Bayesian information criterion (BIC) and likelihood test, as well as the clinical and theoretical rationale were taken into consideration. To identify which model fits our data best while being the most parsimonious, we compared statistical criteria (e.g. BIC) of models with one to six groups. Table 1 presents an overview of the selection criteria regarding different numbers of trajectories.

**Table 1.** Information criteria for models with varying numbers of perinatal depressive symptom trajectory groups (*n* = 151).

Groups	Parameters	BIC	Log likelihood
1	3	5292.186	−2638.567
2	6	5040.492	−2505.194
3	9	4947.644	−2451.244
4	12	4939.978	−2439.885
5	15	4922.184	−2423.462
6	18	4911.706	−2410.698

BIC: Bayesian information criterion.

Furthermore, we used posterior group classification probabilities as a reference for best group model fit. As cases without almost complete data sets (>two time points missing) had already been excluded (*n* = 10), the scattered missing data were imputed before running the GBTM analyses. Missing values in the data were imputed using the non-parametric missing value imputation method missForest.^50^

## Results

### Sample characteristics

Overall, our sample mainly consisted of well-educated, health-conscious Swiss women with a rather high socioeconomic status. On average, the women were 33 years old, with a high level of education (69% with a university degree) and were employed (86.3%). Sample characteristics divided into the four trajectory groups are presented in Table 2.

**Table 2.** Sociodemographic, psychosocial, and behavioral characteristics and group differences of different trajectory groups of perinatal depressive symptoms.

Variable	Trajectory groups of perinatal depressive symptoms					*p-*value
	Total (*n* = 151), *N* (%) ormean (SD)	Low (*n* = 81), *N* (%) or mean (SD)	Medium (*n* = 52), *N* (%) or mean (SD)	High (*n* = 6), *N* (%) or mean (SD)	Pregnancy-only (*n* = 12), *N* (%) or mean (SD)	
Age	32.8 (3.86)	32.7 (3.93)	32.6 (3.68)	35.0 (4.05)	32.4 (4.17)	0.542
Nationality						
Swiss	106 (69.3)	59 (72.0)	36 (69.2)	3 (50.0)	8 (66.7)	0.645
German	25 (16.3)	12 (14.6)	8 (15.4)	2 (33.3)	3 (25.0)	0.583
Austrian	6 (3.9)	4 (4.9)	0 (0.0)	0 (0.0)	2 (16.7)	0.056
Other	23 (15.0)	11 (13.4)	9 (17.3)	2 (33.3)	1 (8.3)	0.519
Education						
Vocational school	20 (13.1)	7 (8.5)	9 (17.3)	2 (33.3)	2 (16.7)	0.221
Higher vocational school	6 (3.9)	2 (2.4)	2 (3.8)	1 (16.7)	1 (8.3)	0.321
General university entrance qualification	5 (3.3)	3 (3.7)	0 (0.0)	0 (0.0)	2 (16.7)	0.035*
University degree	106 (69.3)	62 (75.6)	36 (69.2)	2 (33.3)	6 (50.0)	0.038*
Other	18 (11.8)	10 (12.2)	5 (9.6)	1 (16.7)	2 (16.7)	0.895
None of the above	3 (2.0)	2 (2.4)	1 (1.9)	0 (0.0)	0 (0.0)	0.928
Intention to work after maternity leave						
Yes	49 (32.0)	24 (29.3)	19 (36.5)	3 (50.0)	3 (25.0)	0.605
No	100 (65.4)	56 (68.3)	32 (61.5)	3 (50.0)	9 (75.0)	
Relationship status						0.568
Single	1 (0.7)	0 (0.0)	1 (1.9)	0 (0.0)	0 (0.0)	
In a relationship, but not cohabitating	2 (1.3)	1 (1.2)	1 (1.9)	0 (0.0)	0 (0.0)	
In a relationship and cohabitating	57 (37.3)	30 (36.6)	20 (38.5)	2 (33.3)	5 (41.7)	
Married and cohabitating	88 (57.5)	49 (59.8)	29 (55.8)	3 (50.0)	7 (58.3)	
Married, not cohabitating (separation)	1 (0.7)	0 (0.0)	0 (0.0)	1 (16.7)	0 (0.0)	
Psychiatric illness in family						0.990
Yes	53 (34.6)	27 (32.9)	18 (34.6)	3 (50.0)	5 (41.7)	
No	70 (45.8)	39 (47.6)	26 (50.0)	1 (16.7)	4 (33.3)	
Unknown	28 (18.3)	15 (18.3)	8 (15.4)	2 (33.3)	3 (25.0)	
PPD of a female family member						0.022*
Yes	20 (13.1)	7 (8.5)	9 (17.3)	3 (50.0)	1 (8.3)	
No	131 (85.6)	74 (90.2)	43 (82.7)	3 (50.0)	11 (91.7)	
In psychotherapy						0.795
Yes	16 (10.5)	7 (8.5)	6 (11.5)	1 (16.7)	2 (16.7)	
no	133 (86.9)	73 (89.0)	45 (86.5)	5 (83.3)	10 (83.3)	
Prior depressive symptoms						<0.001**
Yes	42 (27.5)	16 (19.5)	14 (26.9)	5 (83.3)	7 (58.3)	
No	105 (68.6)	62 (75.6)	37 (71.2)	1 (16.7)	5 (41.7)	
Pregnancy planned						0.170
Yes	122 (79.7)	69 (84.1)	41 (78.8)	3 (50.0)	9 (75.0)	
No	29 (19.0)	12 (14.6)	11 (21.2)	3 (50.0)	3 (25.0)	
Delivery mode						0.893
Vaginal	111 (72.6)	58 (70.8)	40 (76.9)	4 (66.7)	9 (75.0)	
C-section	40 (26.2)	23 (28.1)	12 (23.0)	2 (33.4)	3 (25.0)	
Baby nutrition						
Breastfeeding only	114 (74.5)	58 (70.7)	41 (78.8)	6 (100.0)	9 (75.0)	0.409
Combined	34 (22.3)	23 (28.1)	8 (15.4)	0 (0.0)	3 (25.0)	0.456
Formula only	9 (5.9)	4 (4.9)	5 (9.6)	0 (0.0)	0 (0.0)	0.476

SD: standard deviation; PPD: postpartum depression; C-section: cesarean section.

* *p* < 0.05, **p < 0.01.

### Trajectories of perinatal depressive symptoms

**Figure 3.**
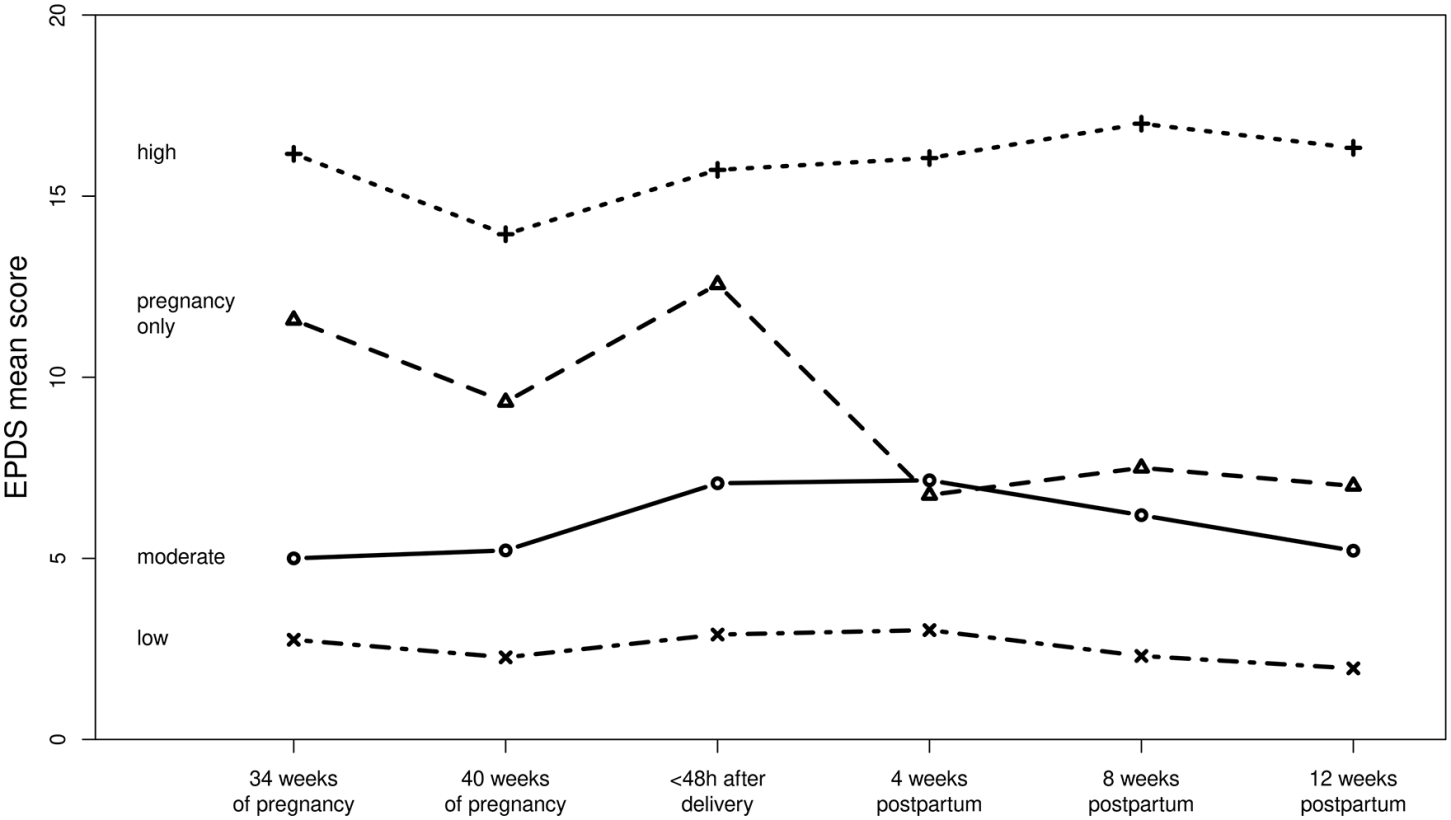
Trajectories of perinatal depressive symptoms.

Although the BIC value decreased as the number of groups increased, from a clinical perspective and to maintain the interpretability of the data, the five- and six-group models do not add additional value, mainly because with an increasing number of groups, group sizes tend to become insignificantly small. Therefore, we chose the four-group model, which showed good average posterior probability classification (>0.80) and overall adequate model fit indices. Regarding the shape of the trajectories, a linear form fitted the data better than a quadratic form. Figure 3 shows the trajectories of the four-group model.

With regard to the trajectory groups, it emerged that the low-symptom group contained the highest number of women (*n* = 81, 53.6%), and comprised women with low depressive symptoms at baseline (34–36 weeks gestation), which remained low throughout pregnancy and the postpartum period. The second largest trajectory group (moderate-symptom group) mostly showed low to moderate depressive symptoms at baseline (*n* = 52, 34.4%), although with a higher mean level than the low-symptom group. Depressive symptoms increased slightly shortly after delivery and remained moderate up to 3 months postpartum. The third group of women (pregnancy-only) exhibited a moderate level of depressive symptoms during pregnancy, which decreased significantly after delivery, returning to a low level in the postpartum phase (*n* = 12, 8%). The fourth and final trajectory group (high-symptom group) comprised women who showed high depressive symptoms at baseline, which persisted to demonstrate a clinically relevant number of depressive symptoms throughout the entire pregnancy and postpartum phase (*n* = 6, 4%). Parameter estimates for each trajectory group can be found in Table 3.

**Table 3.** Parameter estimates, their statistical significance, and standard errors for perinatal depressive symptom trajectories.

Parameters	Trajectory group			
	Low	Moderate	High	Pregnancy-only
Intercept	2.82 (0.27)	5.64 (0.36)	15.18 (0.97)	11.56 (0.68)
Slope	−0.10 (0.09)	0.11 (0.11)	0.29 (0.31)	−0.97 (0.23)**

** *p* < 0.01.

In terms of the clinical relevance of the described perinatal depressive symptoms, some potential limitations of the widely used EPDS cut-off score of 12/13 have recently been discussed. Therefore, we refer instead to the proposed classification that aligns with the Beck Depression Inventory (BDI) instead of using a dichotomous approach.^38^ From this perspective, the severity ranges of depressive symptoms in our sample would indicate no to minimal depression (0–6) for the low-symptom group, mild depression (7–13) for the moderate-symptom group and the pregnancy-only group, and moderate depression (14–19) for the high-symptom group. The mean EPDS scores of all trajectory groups and assessment time points can be found in Table 4.

**Table 4.** Mean (standard deviation) of EPDS scores of different trajectory groups and assessment times.

Trajectory group	Time of assessment					
	34 weeks of pregnancy	40 weeks of pregnancy	<48 h after delivery	4 weeks postpartum	8 weeks postpartum	12 weeks postpartum
Low	2.75 (2.28)	2.02 (2.21)	2.84 (2.98)	2.99 (2.66)	2.26 (2.41)	1.96 (2.39)
Moderate	5.00 (3.21)	5.27 (3.01)	7.06 (4.47)	7.19 (4.12)	6.23 (3.97)	5.21 (3.66)
High	16.17 (2.32)	14.80 (4.38)	16.40 (5.98)	17.00 (5.15)	17.00 (4.73)	16.33 (3.67)
Pregnancy-only	11.58 (3.17)	9.55 (5.03)	12.73 (3.55)	6.75 (3.49)	7.50 (3.85)	7.00 (4.07)

EPDS: Edinburgh Postnatal Depression Scale.

### Predictors of trajectory group affiliation

To evaluate the potential predictive value of psychopathological risk factors, we integrated six relevant factors into the model. Based on the previous literature, the factors of interest were past depression (yes/no), prenatal stress (PDQ), birth anxiety (BAS), quasi-menopausal symptoms after delivery (MRS < 48 h after delivery), state anxiety (STAI), and a history of premenstrual symptoms (PMS). Four out of the six factors emerged as significant for determining group affiliation of perinatal depressive trajectories.

Women with a history of premenstrual symptoms, higher anxiety (birth anxiety and state anxiety), and a higher number of quasi-menopausal symptoms < 48 h after delivery were more likely to be in the moderate-symptom group than in the low-symptom group. In addition, women in the pregnancy-only group and the high-symptom group reported significantly higher scores for state anxiety during pregnancy, more quasi-menopausal symptoms after delivery, and a history of PMS. Regarding prenatal stress, only a trend was observed, insofar as the higher the prenatal stress score, the more likely women were to report depressive symptoms, but only during pregnancy (pregnancy-only group). Contrary to expectations, a history of depression was not a significant predictor of belonging to the high-symptom group.

In sum, a history of PMS, increased anxiety during the third trimester of pregnancy, and quasi-menopausal symptoms after delivery resulted in a greater likelihood of a higher perinatal depressive symptom trajectory. With regard to birth anxiety, a significant association was only found with respect to a switch from the low-symptom to the moderate-symptom group. The results of the analysis are shown in Table 5.

**Table 5.** Psychopathological factors that are associated with trajectory group affiliation of perinatal depressive symptoms.

Variables	Moderate		High		Pregnancy-only	
	AOR	95% CI	AOR	95% CI	AOR	95% CI
Prior depressive symptoms	1.54	0.20–11.81	0.01	5.47e–7–46.06	0.03	2.25e−6–461.45
Prenatal stress (PDQ)	1.02	0.91–1.15	1.48	0.65–3.41	2.05	0.92–4.56
Somatic symptoms (MRS)	1.67**	1.21–2.29	12.11*	1.65–88.16	9.43*	1.29–68.74
Birth anxiety (BAS)	1.09*	1.01–1.19	0.99	0.77–1.30	0.80	0.52–1.23
State anxiety (STAI)	1.21*	1.01–1.43	8.20*	1.19–5.63	7.28*	1.03–5.14
Premenstrual symptoms (PMS)	1.07*	1.01–1.15	2.20*	1.04–4.65	2.14*	1.01–4.56

AOR: adjusted odds ratio; CI: confidence interval; PDQ: Prenatal Distress Questionnaire; MRS: Menopause Rating Scale; BAS: Birth Anxiety Scale; STAI: State-Trait Anxiety Inventory; PMS: premenstrual syndrome.

* *p* < 0.05, **p < 0.01.

## Summary of results

The main findings of this study are as follows. Most mentally healthy women, as well as women with perinatal depressive symptoms, tend to remain rather stable in mood over the course of pregnancy and the postpartum period. Furthermore, we identified new psychopathological risk factors, such as quasi-menopausal symptoms after delivery. Covering an extensive time period of nearly 6 months, we used a group-based trajectory modeling approach to analyze trajectories of depressive symptoms during the peripartum.

We found four trajectory groups of perinatal depressive symptoms, consisting of three relatively temporally stable trajectories that differed in symptom load and one trajectory group with a significant decrease in symptoms postpartum. Half of our sample (53.6%) presented with minimal depressive symptoms throughout the perinatal period, which remained stable over time. A third of the sample experienced a relatively consistent moderate level of depressive symptoms during pregnancy and the postpartum period. Only 4% of women showed clinically relevant, constantly elevated depressive symptoms throughout the peripartum period. While for several women, increased antenatal depressive symptoms decreased significantly after delivery, our analyses did not reveal a subgroup of women with a new onset of depressive symptoms in the postpartum period.

The proposed psychopathological risk factors, which had previously only been validated in cross-sectional studies, proved to be significantly relevant within our comprehensive longitudinal design. In particular, a history of PMS, anxiety, and quasi-menopausal symptoms directly after delivery enhanced the risk of developing perinatal depressive symptoms in subgroups of women.

## Discussion

To date, only a small number of studies have used growth curve mixture modeling approaches to identify trajectories of perinatal depressive symptoms. A major advantage of this modeling approach is the possibility to capture individual differences in perinatal symptom patterns regarding onset, severity, and chronicity of symptoms using a longitudinal design. Instead of treating this population as a homogeneous group, growth curve mixture modeling allows for trajectory modeling without predefining the number of trajectories to secure optimal data fit.^51^ This is especially important from a clinical perspective, as women might present with differing underlying etiopathological mechanisms resulting in distinct symptom profiles. To account for these subgroups, to the best of our knowledge, this study is among the first to specifically include mentally healthy as well as previously or currently depressed women.

The heterogeneity of perinatal depressive symptom patterns was confirmed in our sample, with the analyses revealing four distinct subgroups of women following unique trajectories over 6 months. In line with previous research findings of a subgroup of women showing relevant depressive symptoms only during pregnancy and a sharp symptom decline after giving birth, 8% of the women in our sample exhibited this symptom pattern.^5,6,13,52,53^ In contrast to the results of four previous studies that also used growth curve mixture modeling,^5,6,10,52^ in the present sample, we did not find a group with a new onset of depressive symptoms in the postpartum period. Another study that followed adolescent women during the perinatal period also failed to detect a subgroup with a new onset of depressive symptoms postpartum.^14^ Our findings support existing evidence of a frequent symptom onset during pregnancy, as demonstrated in 53% of women in a recent study by our work group.^20^ Similarly, in a diverse sample from New Zealand, 37% of women showed a symptom onset during pregnancy, compared to only 5% of women postpartum.^54^ Such findings raise the question of whether prevalence rates of antenatal depression are equal to or even higher than rates of postpartum depression. This question has been discussed in the literature and is important for the clinical handling of perinatal depression.^3,10,55^ As proposed by Gavin et al.^3^ the failure to detect the onset of depressive symptoms in the peripartum can be remedied by already providing appropriate screening during pregnancy, with a follow-up in the postpartum period.

With regard to risk factors for perinatal depressive symptoms, most of the existing evidence has been drawn from cross-sectional data,^32^ while longitudinal study designs yield greater predictive value. The few longitudinal studies using growth mixture modeling focused mainly on socioeconomic and psychosocial risk factors, and generally yielded inconclusive findings.^17^ In their review, Baron et al.^17^ found that anxiety, stress, and a lower educational level were risk factors for a high or episodic depressive symptoms trajectory compared to a low depressive symptoms trajectory. The authors emphasized, however, that most of the reported risk factors did not discriminate between high and episodic depressive symptom trajectories, most likely due to homogeneous samples and strict inclusion criteria. In addition, some common risk factors, such as prior or current psychopathological symptoms, which might act as important time-dependent risk factors, have not yet been examined. Nevertheless, evidence from cross-sectional data points to the clinical significance of psychopathological risk factors in terms of increasing perinatal depressive symptoms, and especially factors that are linked to other reproductive transition phases.^23^

In this study, women with a history of premenstrual symptoms showed a significantly heightened risk of being in one of the subgroups of women with a higher symptom load. This finding is in line with the previous literature that demonstrated shared etiological mechanisms between PMS and perinatal depressive symptoms, such as a sensitivity to hormonal fluctuations and genetic vulnerability.^18^

Although a history of depression did not emerge as a significant risk factor in our model, women with clinically relevant perinatal depressive symptoms (EPDS score > 12) were more likely to report prior depression, which was also found in another Swiss sample.^20^ Compared to the other trajectory subgroups found in this study, these women also more often stated that they had at least one female family member with current or past perinatal depression.

Furthermore, women with quasi-menopausal symptoms after delivery were found to be more vulnerable to develop perinatal depressive symptoms. Accordingly, such quasi-menopausal symptoms might act as a moderator, with somatic symptoms such as urogenital complaints, hot flashes, and sleep disturbances triggering or aggravating depressive symptoms in the peripartum.^24,56,57^ Poor sleep seems to be of particular importance regarding the onset and chronicity of perinatal depressive symptoms.^12^ The proposed etiological pathway in this regard is similar to the perimenopause, when these somatic symptoms result from a sensitivity to the inherent hormonal changes.^28,58,59^ A history of PMS, the occurrence of quasi-menopausal symptoms, and the frequently reported family history of perinatal depressive symptoms again emphasize a potential shared etiopathology of reproductive transition phase mood disorders, manifesting in corresponding symptom patterns.

While prenatal stress has been previously mentioned as a relevant risk factor for depressive symptoms throughout the peripartum, our findings only revealed a trend for significance regarding antenatal depression. This is in line with another study, which reported that antenatal depression in particular is strongly associated with perceived stress.^54^

Based on our findings, state anxiety can be added as a significant risk factor, as it emerged as a relevant comorbidity for all perinatal depressive trajectories with the exception of the group of women with minimal symptoms. Three other longitudinal studies that used growth curve mixture modeling found that trait anxiety, being anxious about giving birth, and high worry were risk factors for perinatal depressive symptoms.^5,10,60^ However, Mora et al.^5^ pointed out that being anxious about giving birth was only assessed using one dichotomous variable (yes/no). Two large studies identified anxiety as a highly prevalent comorbidity in perinatal women.^13,15^ In this study, which used a validated self-report questionnaire, birth anxiety was only a significant risk factor for the women with moderate perinatal depressive symptoms. A potential explanation for this finding might lie in the limited significance of birth anxiety compared to long-term risk factors, due to the specific, time-restricted event of giving birth.

In sum, our findings raise important questions about the different longitudinal symptom patterns of perinatal depressive symptoms and their associated risk factors. Even though women with a high symptom load only during pregnancy exhibited all of the proposed risk factors, they bounced back to low levels of depressive symptoms after giving birth, especially compared to the subgroup of women who showed a consistently high symptom load throughout the peripartum. This leads to the question of whether this group of women might benefit from greater biopsychosocial resilience in the postpartum period, such as social support or quicker adjustment to the inherent hormonal fluctuations. An explanation for this finding might lie in the time-limited nature of birth anxiety and prenatal stress, which leads to a reduction in symptom load after delivery. Further research is needed to identify time-dependent risk and resilience factors in order to detect a potential window for intervention in women with a high burden of perinatal depressive symptoms.

## Strengths and limitations

A major strength of this study lies in the scope of assessment time points that were included in the longitudinal design, allowing for a group-based trajectory modeling approach. Another important strength is the assessment of quasi-menopausal symptoms postpartum as a risk factor, which enabled us to evaluate a shared clinical presentation of depressive symptoms during reproductive transition phases, indicating mutual underlying biopsychological mechanisms. To the best of our knowledge, this study is the first to exclusively use the questionnaires that are specific for the peripartum period in a growth mixture model, rather than general self-report measures or dichotomous variables for symptom assessment. Although attrition rates are commonly mentioned as a significant limitation for the longitudinal assessment of perinatal depressive symptoms, we achieved a relatively high retention rate of participants, which was likely due to the ongoing assistance and support provided by the entire study team to prevent dropouts. Moreover, to our knowledge, this study is the first to use a growth mixture model that specifically includes women with current or prior depressive symptoms as well as mentally healthy women.

Several limitations should be taken into account when interpreting the present findings. First and foremost, our sample characteristics might limit the generalizability, as our participants were rather healthy and well-educated. In addition, although growth mixture modeling poses many advantages for data analysis, it entails the possibility of rather small group sizes for some trajectories within its design, thus potentially restricting the statistical power of intergroup differences between groups that comprise only a few individuals. This probably explains why the well-recognized psychopathological risk factor of prior depression was not statistically significant in our model. Sutter-Dallay (2012) also found that a history of self-reported depressive episodes did not influence any of the trajectories of perinatal depressive symptoms, which the authors attributed to small subgroup sizes and limited power, especially for the subgroup with high symptoms (*n* = 16). Although we did use specific questionnaires for perinatal stress, anxiety, and depressive symptoms, it should be noted that all measures were self-report, thus posing a risk of bias.

## Conclusion

The present findings revealed that women follow four different perinatal depressive symptom trajectories, the course of which is influenced by a variety of psychopathological risk factors such as a history of PMS, anxiety, and quasi-menopausal symptoms postpartum. Further research is needed to account for the distinct trajectories of perinatal depressive symptoms in order to meet the unique needs of women who are affected by these symptoms and to provide appropriate prevention, diagnosis, and treatment. A focus on quasi-menopausal symptoms postpartum and their association with depressive mood is essential to gain more insight into the interplay of hormonal fluctuations and depressive symptoms in the peripartum.

## Supplemental Material

sj-docx-1-whe-10.1177_17455057221147391 – Supplemental material for Trajectories of reproductive transition phase mood disorder from pregnancy to postpartum: A Swiss longitudinal study Click here for additional data file. Supplemental material, sj-docx-1-whe-10.1177_17455057221147391 for Trajectories of reproductive transition phase mood disorder from pregnancy to postpartum: A Swiss longitudinal study by Alexandra Johann, Jelena Dukic, Yannick Rothacher and Ulrike Ehlert in Women’s Health

## Declarations

**Ethics approval and consent to participate:** Ethical approval for the study protocol of this study was provided by the Cantonal Ethics Committee Zurich, Health Department, Switzerland, reference number: KEK-ZH-Nr. 2018-02357. Approved written informed consent forms were obtained from each study participant.

**Consent for publication:** Written informed consent for publication was obtained from all participants. The identifying details are only accessible to the authors and are stored safely.

**Availability of data and materials:** The data that support the findings of this study are available from the corresponding author upon reasonable request.
